# Naringin Enhances CaMKII Activity and Improves Long-Term Memory in a Mouse Model of Alzheimer’s Disease

**DOI:** 10.3390/ijms14035576

**Published:** 2013-03-11

**Authors:** Dong-Mei Wang, Ya-Jun Yang, Li Zhang, Xu Zhang, Fei-Fei Guan, Lian-Feng Zhang

**Affiliations:** 1 Department of Pathogen Biology, Medical College, Henan University of Science and Technology, Luoyang 471023, China; E-Mail: wdmzgadyx@163.com; 2 Key Laboratory of Human Disease Comparative Medicine, Ministry of Health, Institute of Laboratory Animal Science, Chinese Academy of Medical Sciences & Comparative Medical Center, Peking Union Medical College, Beijing 100021, China; E-Mails: yangyajun484@hotmail.com (Y.-J.Y.); zhangl@cnilas.org (L.Z.); zhangxu_2011@foxmail.com (X.Z.); guanff@cnilas.org (F.-F.G.)

**Keywords:** Naringin, Alzheimer’s disease, APPswe/PS1dE9 mice, CaMKII

## Abstract

The Amyloid-β (Aβ)-induced impairment of hippocampal synaptic plasticity is an underlying mechanism of memory loss in the early stages of Alzheimer’s disease (AD) in human and mouse models. The inhibition of the calcium/calmodulin-dependent protein kinase II (CaMKII) autophosphorylation plays an important role in long-term memory. In this study, we isolated naringin from *Pomelo peel* (a *Citrus* species) and studied its effect on long-term memory in the APPswe/PS1dE9 transgenic mouse model of AD. Three-month-old APPswe/PS1dE9 transgenic mice were randomly assigned to a vehicle group, two naringin (either 50 or 100 mg/kg body weight/day) groups, or an Aricept (2 mg/kg body weight/day) group. After 16 weeks of treatment, we observed that treatment with naringin (100 mg/kg body weight/day) enhanced the autophosphorylation of CaMKII, increased the phosphorylation of the α-amino-3-hydroxy-5-methyl-4-isoxazolepropionic (AMPA) receptor at a CaMKII-dependent site and improved long-term learning and memory ability. These findings suggest that the increase in CaMKII activity may be one of the mechanisms by which naringin improves long-term cognitive function in the APPswe/PS1dE9 transgenic mouse model of AD.

## 1. Introduction

Alzheimer’s disease (AD) is a degenerative neurological disease that is clinically characterized by progressive cognitive dysfunction [1]. Pathologically, the disease is characterized by the presence of extracellular plaques of Amyloid-β (Aβ), intracellular tangles of hyper-phosphorylated tau protein [2], and forebrain cholinergic neuron loss [3]. The standard treatment for symptomatic AD relies primarily on acetyl-cholinesterase (AChE) inhibitors and Aricept is an example of an AChE inhibitor; only a modest effect on the rate of degeneration is afforded by memantine, a glutamate receptor antagonist [4]. Previous studies have shown that early synaptic dysfunction caused by the accumulation of Aβ may initiate synapse loss, cell death, and cognitive impairment in AD [5,6] and may be fully reversible if the appropriate intervention occurs. A key question to be answered is how Aβ at this stage affects synaptic plasticity in the neuronal networks where memories are formed and stored. Accumulating evidence indicates that calcium/calmodulin-dependent protein kinase II (CaMKII) is a key synaptic target underlying Aβ-induced synaptic depression [7]. Therefore, drugs that enhance CaMKII signaling should be able to oppose Aβ action and rescue synaptic and cognitive function [8]. Thus, there is a need to develop new medications that provide synaptic neuroprotection.

**Figure 1:**
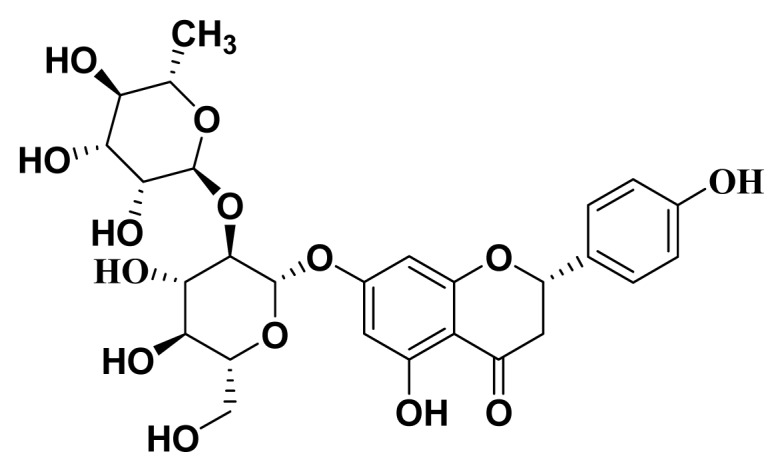
The structure of naringin.

Many plant species containing flavonoids have been widely used in traditional medicine. Recent epidemiological and dietary interventional studies, both in humans and animals, suggest that these flavonoids prevent and delay neurodegeneration, especially in aged-population cognitive dysfunction [9]. Naringin (Figure 1), a well-known flavanone glycoside of *Citrus* fruits, possesses antioxidant, anti-inflammatory, anti-apoptotic, anti-ulcer, anti-osteoporosis and anti-carcinogenic properties [10,11]. Naringin has been reported to attenuate behavioral alterations and cognitive impairment in kainic acid-induced epilepsy models [12] and 3-nitropropionic acid-induced Huntington models [13]. Moreover, naringin administration improves cognitive deficits in colchicines [14] and D-galactose [15] induced learning and memory impairment models. More importantly, it has also been shown that naringin is able to prevent Aβ-induced neurotoxicity *in vivo*[16]. However, little information is available in the literature about the effect of naringin on Aβ-induced long-term cognitive impairment. In this study, we isolated naringin from Pomelo peel and studied the effects of naringin on long-term memory and CaMKII activity in the APPswe/PS1dE9 transgenic mouse model of Alzheimer’s disease.

## 2. Results and Discussion

### 2.1. Naringin Delays the Passive-Avoidance Decline of APPswe/PS1dE9 Mice in a Step-Through Test

**Figure 2:**
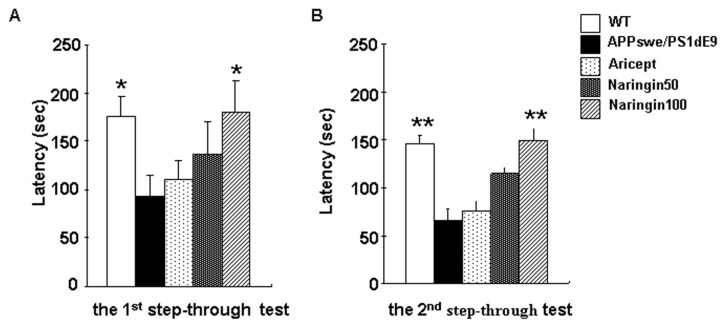
The effect of naringin on long-term cognitive function detected by step-through passive-avoidance test. Non-transgenic littermates (WT), vehicle control (APPswe/PS1dE9), naringin-treated group at a dose of 50 mg/kg/day (Naringin50) or 100 mg/kg/day (Naringin100) and the Aricept-treated group (Aricept) were included. The latency to move into the dark compartment in the 1st step-through test (**A**) and in the 2nd step-through test (**B**) was measured. All data are presented as the mean ± SEM. *p < 0.05, **p < 0.01 compared with APPswe/PS1dE9 mice.

Naringin was isolated from *Pomelo peel*, as described in the methods. We evaluated the effect of purified naringin (purity >98%) on long-term cognitive function. WT mice, untreated APPswe/PS1dE9 mice and APPswe/PS1dE9 mice after 16 weeks of naringin administration underwent two step-through passive-avoidance tests with a seven-day interval. As shown in Figure 2, there was a significant overall group difference in the first latency (*F* (4,45) = 3.07, *p* < 0.05) *versus* the second latency (*F* (4,45) = 4.73, *p* < 0.01) in the two step-through tests among the five groups. A ratio of the decrease of latency ((value of first latency − value of second latency)/value of first latency × 100%) was used to measure long-term cognitive function in the two step-through tests. The ratios of WT group, APPswe/PS1dE9 group, Aricept-treated group and naringin-treated group (100 mg/kg/day) were 15.54%, 34.96%, 31.52% and 17.8%, respectively. Compared with WT mice, the ratio of APPswe/PS1dE9 mice was increased by 125% (*p* < 0.05). Compared with APPswe/PS1dE9 mice, the treatment of naringin and treatment of Aricept reduced the ratios by 49.1% (*p* < 0.05) and 9.8%, respectively.

### 2.2. Naringin Improves Long-Term Learning and Memory of APPswe/PS1dE9 Mice in the Morris Water Maze

**Figure 3:**
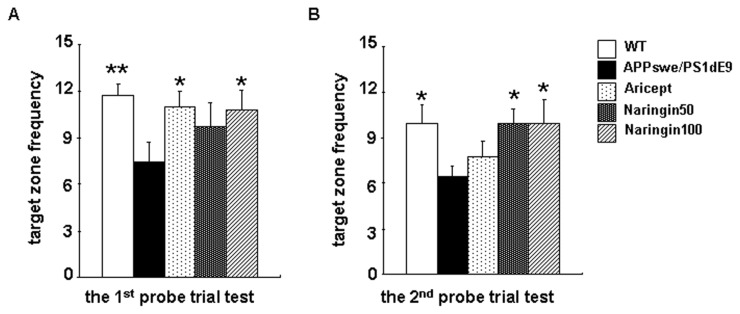
The effect of naringin on long-term learning and memory in APPswe/PS1dE9 transgenic mice using the Morris water maze. The number of target zone crossings in the 1st probe test (**A**) and in the 2nd probe test (**B**) was tabulated. All data are presented as the mean ± S.E.M. *p < 0.05, **p < 0.01 compared with APPswe/PS1dE9 mice.

To examine the effects of naringin on long-term spatial memory, mice underwent two probe tests with a seven-day interval. As shown in Figure 3, there was a significant overall group difference in the frequency of crossing the platform among the five groups in the first probe (*F* (4,45) = 3.08, *p* < 0.05) *versus* the second probe (*F* (4,45) =2.89, *p* < 0.05). A ratio of the decrease of frequency ((value of first frequency − value of second frequency)/value of first frequency × 100%) was used to measure long-term cognitive function in the two probe trials. The ratios of WT group, APPswe/PS1dE9 group, Aricept-treated group and naringin-treated group were 6.43%, 30.1%, 29.27% and 7.58%, respectively. The ratio markedly increased by 368% in APPswe/PS1dE9 mice when compared with WT mice (*p* < 0.01). Compared with APPswe/PS1dE9 mice, the treatment of naringin and treatment of Aricept reduced the ratios by 74.8% (*p* < 0.01) and 2.76%, respectively.

### 2.3. Naringin Enhances CaMKII Activity in an AD Model

**Figure 4:**
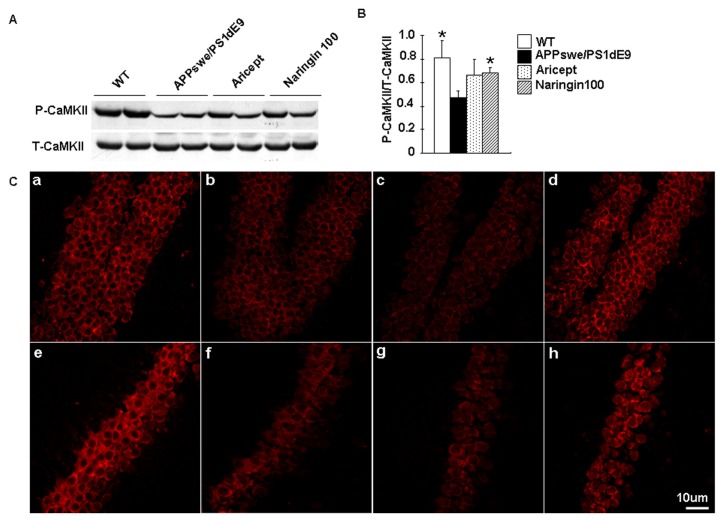
Naringin enhances CaMKII activity *in vivo*. Protein lysates were prepared from the brains of WT mice, APPswe/PS1dE9 mice, Aricept-treated mice and Naringin100-treated mice. The abundance of phosphorylated CaMKII and the total expression of CaMKII were determined by Western blot (**A**). The average blot densitometry of three independent experiments is shown (**B**). Immunostaining for phosphorylated CaMKII in brain slices (**C**) from WT mice (**a**,**e**), APPswe/PS1dE9 mice (**b**,**f**), Aricept-treated mice (**c**,**g**) and Naringin100-treated mice (**d**,**h**). The images are of the dentate gyrus of the hippocampus (top panels **a**–**d**) and the CA1 region of the hippocampus (bottom panels **e**–**h**). All data are presented as the mean ± S.E.M. *p < 0.05, **p < 0.01 compared with APPswe/PS1dE9 mice.

CaMKII is crucial for long-term memory maintenance and is involved in mediating Aβ action at hippocampal synapses. The phosphorylation of CaMKII at Thr286 results in a persistently active form of the kinase that is required for long-term memory. To examine whether naringin enhances CaMKII autophosphorylation and function *in vivo*, we determined the status of CaMKII using antibodies that recognised the phosphorylation of CaMKII at Thr286. Naringin (100 mg/kg/day) treatment increased Thr286 phosphorylation by 47% compared with untreated APPswe/PS1dE9 mice. In contrast, the Aricept treatment did not affect the phosphorylation of CaMKII (Figure 4A,B). There is substantial evidence suggesting that the hippocampus plays a central role in memory functions via interconnections with distributed cortical regions [17]. To examine whether CaMKII activity was altered in the hippocampus, brain sections from WT, untreated APPswe/PS1dE9 mice and naringin-treated APPswe/PS1dE9 mice were stained with an antibody against p-CaMKII. Increased CaMKII phosphorylation was evident in naringin-treated mice (Figure 4C), suggesting that naringin (100 mg/kg/day) induced the activation of CaMKII, predominantly in the hippocampus.

AChE inhibition alleviates the symptoms of AD but has only a modest beneficial effect on neurodegeneration [4]. In this study, Aricept showed a short duration of action on memory ability, indicated by a 31.52% decrease in the second latency and 29.27% decrease in the second frequency compared with the first in the passive-avoidance task. However, the naringin-treated (100 mg/kg/day) group exhibited long-term effects on memory ability, indicated by only a 7.58% decrease in the second frequency and 17.8% decrease in the second latency. Therefore, we have shown that naringin improved the long-term cognitive function in APPswe/PS1ΔE9 transgenic mice.

The Aβ-induced impairment of hippocampal synaptic plasticity is considered an underlying mechanism for memory loss in the early stages of Alzheimer’s disease [18]. It has been shown that Aβ prevents the activation of CaMKII and subsequent phosphorylation of α-amino-3-hydroxy-5-methyl-4-isoxazolepropionic (AMPA) receptors [19]. CaMKII is essential for synaptic plasticity and cognitive function [20]. The autophosphorylation at Thr^286^ of CaMKII is known to result in persistent activity and promote translocation of the kinase to the postsynaptic density [21]. This process enables CaMKII to modulate multiple synaptic proteins in a highly efficient manner and thus enhances LTP. Presynaptically, CaMKII-mediated phosphorylation of synapsin 1 promotes its dissociation from synaptic vesicles, causing increased neurotransmitter release [22]. At the postsynaptic site, the best known substrate of CaMKII is the AMPA-type glutamate receptor. CaMKII-induced phosphorylation of AMPA receptors at Ser^831^ of the GluR1 subunit increases the channel conductance of the receptor [23], which can directly contribute to LTP expression. Aβ reportedly reduces the number of synaptic AMPA receptors [24], blocks activity-dependent AMPA receptor phosphorylation [25] and may cause the loss of dendritic spines in the hippocampus [26]. Our previous study has shown the reduction of Aβ by naringin in the APPswe/PS1dE9 transgenic mouse model of AD. The activation of postsynaptic CaMKII is also known to trigger rapid delivery of AMPA receptors from non-synaptic sites into synapses [27] and promote the activity-dependent growth of dendritic spines and formation of synapses [28]. Activation of CaMKII and inhibition of Glycogen synthase kinase (GSK)-3β are both observed in synaptic plasticity [29,30]. Study indicates that CaMKII regulates neuronal survival through the inhibition of GSK-3β activity and GSK-3β is therefore a key candidate to mediate other biological effects of CaMKII [31]. Consistent with this, we observed inhibition of GSK-3β activity and enhancement of CaMKII activity in narigin-treated mice brain, suggesting the crucial role of CaMKII/GSK-3β pathway in neuro-protective effects of naringin. Taken together, the positive effect of naringin on long-term memory improvement might profit from the enhancement of CaMKII activity and/or the reduction of Aβ-induced synaptic dysfunction. It should be mentioned, however, that our study does not exclude the possibility that naringin can act through other signaling pathways to rescue synaptic function. Future studies are needed to determine the involvement of these pathways in the protective effect of naringin.

## 3. Experimental Section

### 3.1. Isolation of Naringin

Naringin was isolated and purified from *Pomelo peel* (a *Citrus* species) according to a previously described method [32]. Briefly, the dry powdered peel was macerated in methanol for 3 days. The slurry was filtered, and the obtained methanolic extract was dried with a rotary evaporator under reduced pressure. The dry methanolic extract was dissolved in water, and dichloromethane (15%) was then added. The mixture was swirled and left for 4 days at room temperature. The crystals were collected by filtration. The substance was re-crystalised to be identified as naringin. Before use, the structure of naringin was confirmed using 1H-NMR and ESI-MS analyses, and its purity was higher than 98%, as determined using HPLC. 1H-NMR (DMSO-*d*_6_, ppm): δ 12.07 (s, 1H), 9.62 (s, 1H), 7.34 (d, *J* = 8.0 Hz, 2H), 6.82 (d, *J* = 8.0 Hz, 2H), 6.13 (s, 1H), 6.11 (s, 1H), 5.55–5.49 (m, 1H), 5.32 (d, *J* = 5.0 Hz, 1H), 5.17–5.12 (m, 3H), 4.73 (d, *J* = 4.5 Hz,1H), 4.67 (d, *J* = 4.5 Hz, 1H), 4.59–4.57 (m, 1H), 4.48 (d, *J* = 5.5 Hz, 1H), 3.74–3.66 (m, 3H), 3.50–3.42 (m, 4H), 3.39–3.31 (m, 2H), 3.24–3.18 (m, 2H), 2.77–2.71 (m, 1H), 1.17 (d, *J* = 6.0 Hz, 3H). ESI-MS (negative, *m*/*z*): 579.2 [M − H]^−^, 1159.1 [2M − H]^−^.

### 3.2. APPswe/PS1dE9 Mice and Naringin Treatment

In this study, we used APPswe/PS1dE9 mice (C57/BL) that were generated as previously described [16]. The APPswe/PS1dE9 mice were bred in an AAALAC-accredited facility. The use of animals was approved by the Animal Care and Use Committee of the Institute of Laboratory Animal Science of Peking Union Medical College.

Three-month-old male and female APPswe/PS1dE9 transgenic mice were randomly assigned among treatment groups. APPswe/PS1dE9 transgenic mice were fed either AIN-76A chow (Dyets Inc., Bethlehem, PA, USA) containing a low dose of naringin (50 mg/kg/day; *n* = 10), a high dose of naringin (100 mg/kg/day; *n* = 10), a dose of Aricept (2 mg/kg/day; *n* = 10) or no drug (*n* = 10) for 16 weeks before being killed. Non-transgenic littermates were fed chow as wild type (*n* = 10). Each group included five males and five females. The dose of naringin was selected based on previous studies [16]. Aricept, an AChE inhibitor, is currently used for the long-term treatment of patients with AD. The dose of Aricept administered was calculated from the weight of the mice to be equivalent to the human dose. Upon conversion of animal dose to the equivalent human dose (human dose (mg/kg) = mouse dose (mg/kg) × (3/37)) [33], a dose of 0.2 mg/kg/day Aricept in humans corresponded to 2 mg/kg/day in mice.

### 3.3. Passive-Avoidance Test

The apparatus for the step-through passive-avoidance test consisted of an illuminated compartment (100mm × 120mm × 100 mm, with a light at the top of the compartment (27 W, 3000 lx)) and a dark compartment of the same size separated by a wall with a guillotine door. In the training session, each mouse was trained to adapt to the step-through passive-avoidance apparatus. The animal was put into the illuminated compartment, facing away from the dark compartment, and was allowed to move into the dark compartment freely for 3 min/day for 3 consecutive days. The learning session of the 4th day was similar to the adaptation trials, except that the door was closed automatically as soon as the mouse completely entered the dark compartment (4-paw criterion) and received a mild foot shock (0.5 mA, 2 s) delivered through the grid floor. The mouse was kept in the chamber for 10 s before being returned to its home cage. On the next day, the mice were placed in the lit compartment for the first test, and the latency to move into the dark compartment and the number of times the mice stepped into the dark compartment was measured for 300 s. To examine the effects of naringin on long-term cognitive function, mice were again placed in the lit compartment, and the latency was recorded on the 7th day after the first test.

### 3.4. Morris Water Maze

Spatial learning and memory was tested using the Morris water maze, performed one day after the end of the open-field test. The protocol for the Morris water maze test was modified from previously reported methods [34]. Briefly, the apparatus included a pool with a diameter of 100 cm that was filled with opaque water at approximately 22 ± 1 °C. An escape platform (15 cm in diameter) was placed 0.5 cm below the water surface. Geometric objects with contrasting colours were set at the remote ends of the water tank as references. The room temperature was constant, and the lighting was even throughout the room. Spatial memory was assessed by recording the latency time for the animal to escape from the water onto a submerged escape platform during the learning phase. The mice were subjected to four trials per day for 5 consecutive days. The mice were allowed to stay on the platform for 15 s before and after each trial. Twenty-four hours after the learning phase, mice swam freely in the water tank without the platform for 60 s. The time spent in the region of the original platform and number of passes through the quadrant and region of the platform were recorded.

To examine the effects of naringin on long-term spatial memory, mice underwent a second test on the 7th day after the initial test. Performance was monitored with a video tracking system (Noldus Ltd., Ethovision XT, Wageningen, The Netherland).

### 3.5. Tissue Preparation

Following the behavioral test, mice were randomly chosen and deeply anesthetised with sodium pentobarbital (100 mg/kg intraperitoneally; *n* = 4). Brains were removed and dissected through the mid-sagittal plane. One hemisphere was immediately sectioned coronally at 8 μm on a freezing microtome (LEICA CMl850, Nussloch, Germany). The sections were fixed with 4% paraformaldehyde for 20 min and then immersed in 0.01 M PBS for 30 min, followed by ethanol for 2 min. Sections were stored at −20°C until immunostaining was performed. The remaining hemibrains were directly homogenised in RIPA buffer containing 0.1% PMSF and 0.1% protease inhibitor cocktail (Sigma-Aldrich, St. Louis, MI, USA). The lysates were centrifuged at 14,000 rpm for 30 min at 4 °C. The protein concentration in supernatants was determined using the BCA method. The protein was stored at −80 °C until a Western blot was performed.

### 3.6. Western Blot Analysis

Equal amounts of soluble protein were separated by SDS-PAGE and transferred onto a nitrocellulose membrane (Immobilon NC; Millipore, Molsheim, France). Immunoblotting was carried out with antibodies specific for p-CaMKII (Thr^286^) (1:1000, Abcam), CaMKII (1:1000, Abcam), p-GluR1 (Ser^83l^) (1:500, Abcam) and GluR1 (1:500, Abcam). Primary antibodies were visualized with anti-rabbit HRP-conjugated secondary antibodies (Santa Cruz Biotechnology, Inc., Santa Cruz, CA, USA) using a chemiluminescent detection system (Western blotting Luminal Reagent; Santa Cruz Biotechnology, Inc.). Variations in sample loading were corrected by normalizing to GAPDH levels.

### 3.7. Immunofluorescence Staining

The brain sections were incubated in 0.01 M PBS containing 0.3% Triton X-100 (PBST) for 20 min at room temperature (RT) and then blocked for 30 min at RT in 8% normal goat serum (ZSGB, Beijing, China). Sections were incubated overnight at 4 °C in a mixture of primary antibodies (p-CaMKII 1:1000, Abcam). After a rinse with 0.01 M PBS, immunoreactivity was visualized by incubation with appropriate secondary antibodies conjugated to red fluorescent TRITC (ZSGB, Beijing, China). After a thorough rinse with 0.01 M PBS, the cover slips were mounted on slides using a fluorescence mounting medium (ZSGB, Beijing, China). The results of staining were visualized using an inverted fluorescence microscope (Olympus, Tokyo, Japan) with an excitation wavelength of 555 nm for TRITC (red). Images were captured using ProgRes microscope cameras (Zeiss, Jena, Germany).

### 3.8. Statistical Analysis

All data were expressed as the mean ± SEM. The latency of entering the dark compartment in the step-through test, as well as the frequency of platform area crossing in the probe trials of the Morris water maze, were analyzed by one-way ANOVA, followed by LSD (equal variances assumed) or Dunnett’sT3 (equal variances not assumed) for a post hoc test between groups. The difference between the first latency/frequency and the second latency/frequency were analysed with a paired ANOVA test. The proteins were analyzed by one-way ANOVA, followed by LSD. All analyses were performed with the SPSS statistical package (version 13.0 for Windows, SPSS Inc., Chicago, Illinois, IL, USA, 2004). Differences were considered significant at a *p*-value < 0.05.

## 4. Conclusions

In conclusion, naringin improves the long-term memory ability in an AD transgenic mouse model. Enhancement of CaMKII activity may be the primary mechanism by which naringin affects long-term cognitive functions in AD. To our knowledge, this is the first report of improved long-term memory with naringin treatment in AD. Naringin shows therapeutic promise in an AD transgenic mouse model, and it may have potential as an AD therapeutic agent.
